# Rheumatism and Tuberculosis

**Published:** 1913-02

**Authors:** R. H. L. Bibb

**Affiliations:** Austin, Texas


### TEXAS MEDICAL JOURNAL.

Established July, 1885

F. E. DANIEL, M. D., - - - - - Editor, Publisher and Proprietor

Published Monthly.—Subscription, $1.00 a Year.

VOL XXIX. AUSTIN, FEBRUARY, 1913. No. 8.

The publisher is not responsible for the views of the contributors.

### Original Articles.

For Texas Medical Journal.

### Rheumatism and Tuberculosis

BY R. H. L. BIBB, M. D., AUSTIN, TEXAS.

For many years the profession has regarded rheumatism and tuberculosis as antagonistic—the presence of one assuring immunity to the other—and this opinion was recently very strongly accentuated by one of the creators of medical faith declaring that rheumatics are resistant to the inroads of tuberculosis, and that the tubercle bacilli cannot flourish in a rheumatic soil.

Notwithstanding this authoritative assertion, and this faith, the latter somewhat sanctified by age, there are those, chiefly of the modern French school, irreverent and iconoclastic enough to profess to see a no remote kinship between the two diseases—a "something in common," and sometimes the one displacing or merging into the other.

According to these teachings, rheumatism may be inherited and acquired. The heirs to rheumatism manifest, in early life, this *predisposition* by gastro-intestinal, cutaneous and asthmatic symptoms, whilst those who acquire it, after passing infancy and childhood free from arthritic indications, and after a life of sedentary indulgence in the luxuries of the table, malted and distilled liquors, find themselves, somewhere between 40 and 50, the victims of vicious nutrition. They become confirmed rheumatics.

It is not claimed that rheumatic heirs are born with perturbed nutrition, renal and hepatic insufficiency already installed, but that they come with a *predisposition*—with the soil prepared—to a faulty metabolism and to auto-intoxications which incline them to contract the diseases of their ancestors—as in tuberculosis—with greater facility and in greater numbers than occurs with others not thus constituted. And, inexplicable as it is, this *predisposition* is very greatly more marked when paternal than when maternal—which is, doubtless, true of other diseases also; but when it is double, i. e., when it converges upon the offspring from both father and mother, it becomes still more marked, as is seen, also, in consanguineous marriages in which neither of the parents had manifested any stigmata of rheumatism.

There are families in which rheumatism and tuberculosis appear with equal frequency in both the old and the young. From this fact arises the claim that, although at first sight these pecancies appear widely separated, they are, in fact, in relation to cause and effect, very closely allied—so close, indeed, that Poncet declares that "tuberculosis and rheumatism have the same origin."

The tubercular—contrary to what takes place in the rheumatic—are in a condition of hyper-metabolism, and tissue waste occurs with great rapidity, especially in the first and second stages of the disease. There is rapid loss of the mineral salts, the fats are quickly consumed, and they soon become greatly reduced in weight, strength and bulk. Their temperature runs high and is generally increased by exercise and fatigue. They improve with rest, fresh air, forced alimentation, tonics, especially preparations of arsenic and the alkaline minerals—in short, with just such measures as tend to the production of arthritism.

Emphysema—essentially of rheumatic origin—is frequently one of the most valuable and one of the earliest symptoms that a given case of pulmonary tuberculosis has recovered; and it is a matter of daily observation, wherever autopsies are made on those dying of other diseases, that the spontaneous cure of pulmonary tuberculosis is brought about, or is accompanied by, calcifition of the tubercular foci in the lungs.

With the rheumatic these facts are antithetic. With them the temperature is habitually subnormal; metabolism is imperfect and sluggish; they tend to obesity; exercise is beneficial; repose, hyper-alimentation, fats and arsenicals are highly prejudicial. They show a marked proneness to sclerotic and fatty degeneration of their excretory organs and to the formation of bladder, nephritic and biliary calculi. Methods addressed to their relief would be rapidly fatal to the tubercular. Besides, it is claimed that the *bacillus tuberculosa* "will not flourish in rheumatic soil."

From the foregoing rather incomplete contrast, at first sight, the antagonism between rheumatism and tuberculosis would appear to be undeniable, but the iconoclasts claim there are other facts which prove they are not only not antagonistic, but are, in many cases, if not in all, actually one and the same thing. The production of localized and general adiposis by injections of attenuated cultures of Koch's bacillus as practiced by Carnot; the many tubercular who suffer from cystic, nephritic and hepatic stones; the observations of Mousseaux of many tubercular who have been cured by improved hygiene, repose and forced feeding becoming rheumatics; the many children of the rheumatic with enlarged glands and other tubercular symptoms gradually becoming arthritics as fast as they are relieved of tuberculosis, as pointed out by Le Gendre; the declarations of De Fleury, who asserts he has often seen unmistakable symptoms of tuberculosis in the arthritic, and that nothing is more common in rheumatism than latent tuberculosis, may be cited in passing.

In the opinion of Poncet and Leriche, rheumatism is an inflammatory neoplasia with a fibrotic and a caseous tendency, fibrotic when arthritic and caseous when tubercular; that lesions often called rheumatic are frequently no more nor less than a local expression of benign tuberculosis, and that the habitual use of the term should be retained for no other purpose than to indicate a soil vaccinated with tuberculosis against a malignant form of that disease. De Fleury says it is not infrequent to meet with obese cases of rheumatic *predisposition* who have been taking thyroid preparations, underfeeding and overexercising, by directions of their physicians, to reduce their size and weight, who some morning expectorate sputa charged with tubercle bacilli. He claims to have recently seen seven such cases.

Arthritism is inherited and is coextensive with humanity; tuberculosis is equally extensive, perpetuates its soil also by inheritance and is contagious, the germ of which, at times harmless or nearly so, at others virulent, is a constant guest of the human family—so constant that autopsies of persons dying of other diseases show it has been present in 50 pro 100 in such cases and, according to Bouchard, in 97 pro 100 in those dying after the age of 70 years, although the combined mortality from tuberculosis ranges between 15 and 20 pro 100.

Germany, a nation which takes the best of care of its soldiers, exacts military service from all of its able-bodied young men between certain ages and for a specified time, sending the best to serve in the Imperial Guard at Berlin—a corps which boasts of the flower of the German empire, and naturally of the most resistant to microbic and other infections.

Several years ago a military surgeon subjected this array of vigorous young men to the cuti-reaction, with the result that *four-fifths* of this flower (if the writer is not mistaken) gave a positive reaction. Of course, it would be erroneous to suppose all of this number, though stamped with the stigmata of the disease, developed active tuberculosis during military life or after returning to civil employment. It is known, however, without writing in specific numbers, that many who resumed manual labor amid improper hygiene and insufficient food died, those who gave themselves to indolency, drink and immoderate eating became rheumatic, whilst those who lived rationally enjoyed immunity from both diseases. The two latter classes, according to our iconoclasts, may be regarded as cases of cured tuberculosis and the penultimate as examples of tuberculosis evolutionizing, under favorable environments, into arthritism.

The writer is not sitting in judgment upon this question. He is content with merely calling attention to the newer views on the subject whilst holding fast to the old until more convincing proof than that detailed above is at hand, fully aware that not infrequently our faiths of today become the objects of our "fleer and gibe and laugh and flout" of tomorrow, not forgetting that Professor Poncet, of Lyons, asserts that "tuberculosis and arthritism have the same origin and that they are one and the same thing." Is it true?

---

When operating upon a carcinoma of the stomach, be sure to suture the layers of the abdominal wall with silk or linen. In these cases healing is very slow and the wound may burst open if absorbable sutures are used.—*American Journal of Surgery.*

---

Circumscribed tenderness is especially significant of the location of a foreign body. Making allowance for the tenderness due to infection, when this is present, a point of persistent maximum tenderness is fairly diagnostic of the location of the body beneath that point. By pressure, with the finger tip or a slender instrument, on one spot after another in the suspected region, one may elicit only a single point of tender, or a point of maximal, and several adjacent points of lesser tenderness. The single or maximal point indicates usually the location of the most superficial part of the foreign body, especially if it be a needle or sharp splinter of glass, wood, etc.; the points of lesser tenderness map out, in a rough way, the general direction of the body.—*American Journal of Surgery.*

